# COVID-19 vaccine uptake and predictors of hesitancy among healthcare workers in Côte d’Ivoire

**DOI:** 10.4102/jphia.v16i1.678

**Published:** 2025-04-24

**Authors:** Richard B. Yapi, Guillaume B.Y. Zamina, Martial Bama, Yao M.R. Amani, Francis Kakooza, Suzan Nakasendwa, Tonny Muwonge, Rodgers R. Ayebare, Leah Mbabazi, Agnes Kiragga, Senga Sembuche, Elizabeth Gonese, Tamrat Shaweno, Nebiyu Dereje, Mosoka P. Fallah, Tajudeen Raji, Issaka Tiembré

**Affiliations:** 1 Department of Research and Development, Centre Suisse de Recherches Scientifiques en Côte d’Ivoire, Abidjan, Côte d’Ivoire; 2 Centre d’Entomologie Médicale et Vétérinaire, Université Alassane Ouattara, Bouaké, Côte d’Ivoire; 3 National Institute of Public Health (NIPH), Abidjan, Côte d’Ivoire; 4 Department of Global Health Security, Infectious Diseases Institute, Makerere University, Kampala, Uganda; 5 Africa Population and Health Research Centre, Nairobi, Kenya; 6 Africa Centers for Disease Control and Prevention, Addis-Ababa, Ethiopia

**Keywords:** Côte d’Ivoire, COVID-19, vaccine, uptake, hesitancy, healthcare workers

## Abstract

**Background:**

Vaccine hesitancy hinders COVID-19 control, especially among healthcare workers (HCWs).

**Aim:**

This study examined factors influencing COVID-19 vaccine uptake and hesitancy among HCWs in Abidjan, Côte d’Ivoire.

**Setting:**

The study was conducted among healthcare workers in Abidjan, the capital city of Côte d’Ivoire.

**Methods:**

A cross-sectional study was conducted from May 2023 to June 2023 in Abidjan. A total of 240 HCWs completed a questionnaire on vaccination attitudes, hesitancy factors and willingness to recommend vaccines. Descriptive statistics and modified Poisson regression estimated adjusted prevalence ratios (aPR) at a 95% confidence interval.

**Results:**

Among participants, 57.5% were female, with a median age of 40 years (IQR: 33–45). HCWs included physicians (26.7%), nurses/midwives (22.5%) and pharmaceutical staff (19.2%). They worked in teaching hospitals (23.3%), general hospitals (30.8%) and community hospitals (45.8%). Vaccine uptake was 73.3%, with 53.3% fully vaccinated and only 4.6% receiving a booster dose. However, 42.1% exhibited vaccine hesitancy, mainly due to concerns about side effects (52.2%). While 55.0% would recommend the vaccine, only 46.3% felt confident addressing patient questions. Age was positively correlated with vaccine uptake: HCWs aged 35–44 years, 45–54 years and 55–65 years were 1.60, 1.68 and 1.78, respectively times more likely to be vaccinated, respectively, compared to those aged 22–34 years.

**Conclusion:**

Vaccine hesitancy (25%) and low booster uptake (4.6%) highlight the need for targeted education and pharmacovigilance. Strengthening HCWs vaccine knowledge and trust is essential for epidemic control.

**Contribution:**

This study underscores the importance of Ministry of Health-led interventions to improve HCWs vaccination rates in Africa.

## Introduction

The successful prevention of infectious diseases hinges on having a successful vaccine that is both accessible and widely accepted. Undoubtedly, vaccines have played a crucial and life-saving role in public health.^1^ Over the past four decades, vaccination programmes in Africa have achieved remarkable advancements.^2^ Effective vaccines have made it possible to prevent and control diseases such as yellow fever, meningitis, chickenpox, tetanus, hepatitis, poliomyelitis and many others.^3^ Despite these achievements, a growing number of individuals in Africa are hesitant to undergo vaccination, both for themselves and their children, thereby undermining the progress made thus far.^4^

The COVID-19 pandemic has profoundly disrupted the psychosocial and economic well-being of people and healthcare systems worldwide. In response to the rapid transmission of the virus during the acute phase and the ensuing health impact, urgent measures focused on non-pharmaceutical interventions, including social distancing and mask use in public, were implemented in the country. The aim of such measures was to mitigate the spread of the virus, protect vulnerable populations and alleviate the strain on healthcare systems, while awaiting the development of an effective vaccine.^5^

When vaccines were made available to combat this deadly virus, Côte d’Ivoire actively participated in the COVID-19 Vaccines Global Access (COVAX) initiative, led by Gloabl Alliance for Vaccines and Immunization (GAVI), the Vaccine Alliance, the World Health Organization, United Nation International Children’s Emergency Fund (UNICEF) and the Coalition for Innovations in Epidemic Preparedness. As one of the early participants, the country received its first doses of the COVID-19 vaccine in February 2021. In the distribution of the vaccine, priority access was granted to vulnerable populations, including healthcare workers (HCWs), who were at the forefront of the battle against COVID-19.^6,7^ However, the uptake of COVID-19 vaccines initially faced various challenges, including the widespread vaccine misinformation. This prompted concerted efforts to address this obstacle and enhance vaccine coverage across diverse communities, particularly for HCWs.^8^

Indeed, HCWs play a crucial role in the response to pandemics, not only as frontline caregivers but also as trusted members of their respective communities. Their vaccination is essential to the vitality of the healthcare system for a number of reasons. It enables them to continue providing care without being agents of disease transmission and to protect their vulnerable patients. Above all, as visible role models, vaccinated HCWs can earn the public’s trust and encourage their communities to get vaccinated. Their reluctance or refusal to do so could fuel public scepticism and resistance, undermining public health efforts.

In May 2023, the World Health Organization announced that COVID-19 is no longer considered a global public health emergency.^9^ Nevertheless, protecting high-risk populations, including HCWs, the elderly and individuals with comorbidities, remains a critical priority in adapting to the new reality. This study aimed to assess the extent of COVID-19 vaccine acceptance among Ivoirian HCWs and identify factors contributing to vaccine hesitancy within this group. The findings could help inform policy and public health strategies around vaccine communication and strengthen health system trust to prevent future pandemics of this kind.

## Research methods and design

### Study design

This was a cross-sectional study conducted among healthcare personnel aged 18 years and above.

### Setting

The study was conducted from May 2023 to June 2023 in ten health facilities in Abidjan, Côte d’Ivoire, across the three levels of the health pyramid, which has the country’s highest population density.

### Study population and sampling strategy

The study utilised a purposive sample of HCWs. Participants were recruited from HCWs in healthcare facilities situated in the health districts of Treichville-Marcory in the South-East and Cocody-Bingerville in the North of Abidjan, known as the epicentres of the COVID-19 epidemic in Côte d’Ivoire.

**FIGURE 1:**
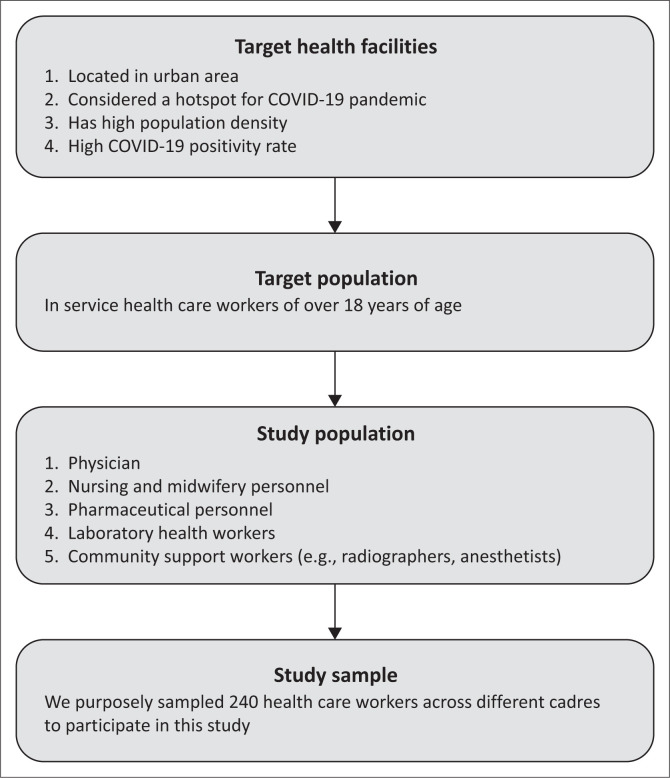
Diagram showing study compliance from a cross-sectional survey conducted in ten healthcare facilities in Abidjan, Côte d’Ivoire, between May 2023 and June 2023.

Figure 1 depicts the study diagram. The recruitment strategy aimed to enlist 200 participants across diverse healthcare professions, selecting them by cadre and in proportion to their regular patient interactions and potential leadership roles within teams. The inclusion criteria were as follows: (1) currently employed in health care work, (2) aged 18 years or older, (3) willing to participate in the study and (4) provided written informed consent. This approach also took into account the variety and geographic spread of healthcare institutions. The composition included physicians (*n* = 60), nursing and midwifery personnel (*n* = 40), pharmaceutical personnel (*n* = 20), laboratory health HCWs such as radiographers and anaesthetists (*n* = 40), and community support and public health officers (*n* = 20).

### Study design and data collection procedures

The list of active HCWs was obtained from the human resources directorate of each selected healthcare facility to verify that selected participants are indeed staff members of the respective healthcare facility. Subsequently, research assistants contacted HCWs by cadre, who expressed willingness to participate. Upon obtaining signed written consent, the research assistants administered a structured questionnaire to assess the adoption, willingness, attitudes and obstacles regarding COVID-19 vaccines. The questionnaire was developed after the behavioural and social drivers model for creating vaccine demand. The questions covered the four principles of thinking and feeling, social processes, motivation and practical issues.^10^ The questionnaire had 17 questions of which 16 were directly related to vaccine confidence and uptake and the last question focused on the sources of information about COVID-19 vaccines used and trusted by HCWs. The questionnaire was piloted with HCWs from the National Institute of Public Health, who were not the target population of the study.

Data entry was carried out directly into Case Report Forms (CRFs) using the Research Electronic Data Capture (REDCap) tool hosted at the Africa Centres of Disease Control and Prevention (Africa CDC), utilising mobile devices. Research assistants ensured real-time data entry to guarantee completeness and accuracy before periodically uploading them into the REDCap database, ensuring the high quality of the collected data. The database was protected and access to the REDCap platform was restricted to the research team through password-protected accounts. Prior to implementing the study, all data collection tools underwent pre-testing to ensure their effectiveness.

### Data analysis

The data were exported from REDCap, and statistical analyses were conducted using Stata version 18 (Stata Corp., College Station, Texas, United States [US]). Descriptive statistics for barriers and enhancers of COVID-19 vaccine uptake were obtained as frequencies and percentages. Vaccine uptake was categorised into four groups including participants who had reported not having received a COVID-19 dose, those who had received a part of the full COVID-19 vaccine schedule, those who had received the full COVID-19 schedule and those who had received additional dose(s) beyond the full primary COVID-19 vaccine schedule (boosted). The comparison of frequency responses was carried out using the Chi-square test or Fisher’s exact test, as appropriate. To assess the association between demographic characteristics and COVID-19 vaccine uptake, a modified Poisson regression model with robust standard errors for multivariable analysis was computed and expressed as an adjusted prevalence ratio (aPR) for gender, HCW cadre, health facility, HCW’s confidence in COVID-19 vaccines and willingness to recommend the vaccine, and age, as described by Greenwood and Yule in 1920.^11^ The Poisson model is used to count data for events occurring independently. The model assumes that events occur independently, meaning the occurrence of one event does not influence the likelihood of another. It also assumes that events happen at a constant average rate over a fixed period or space. Finally, the model requires the mean and variance of the distribution to be equal. This model was employed to analyse the relationships with vaccination status (vaccinated *vs* unvaccinated), given that the percentage of the vaccinated exceeded 15%. Individuals were classified as vaccinated if they had received a single dose of a one-dose vaccine, both doses of a two-dose vaccine or a booster dose. Those who did not meet these criteria were classified as unvaccinated. The significance level was 0.05 and the confidence interval (CI) was 95%.

### Ethical considerations

Ethical clearance for the study was acquired from the National Committee of Ethics of Life Science and Health (CNESVS) under the Ministry of Health, Public Hygiene and Universal Health Insurance (reference number 003-23/MSHPCMU/CNESVS-KM). In addition, the General Directorate of Health granted permission to conduct the study and access healthcare facilities. Finally, written informed consent was obtained from each participant, ensuring their voluntary participation and understanding of the study’s objectives and procedures.

## Results

### Participants’ characteristics

Table 1 outlines the characteristics of the participants with their vaccination status. Overall, 240 individuals provided written informed consent and successfully completed the questionnaire. The age range of participants spanned from 22 years to 65 years, with a median (interquartile range [IQR]) age of 40 (33–45) years. Female participants constituted a larger proportion than males, with 57.5% females compared to 42.5% male participants.

**TABLE 1:** Demographic characteristics of healthcare workers recruited in ten healthcare facilities in Côte d’Ivoire.

Characteristics	Frequency (*n* = 240)				Vaccinated† (*n* = 139)				Unvaccinated (*n* = 101)				*p*
	*n*	%	Median	IQR	*n*	%	Median	IQR	*n*	%	Median	IQR	
**Age in years**	-	-	40	33–45	-	-	42	36–48	-	-	36	31–43	-
18–24	4	1.7	-	-	3	2.2	-	-	1	1.0	-	-	< 0.001
25–34	64	27.5	-	-	24	17.3	-	-	42	41.6	-	-	-
35–44	106	44.2	-	-	68	48.9	-	-	38	37.6	-	-	-
45–54	49	20.4	-	-	31	22.3	-	-	18	17.8	-	-	-
55–65	15	6.3	-	-	13	9.4	-	-	2	2.0	-	-	-
**Gender**													
Female	138	57.8	-	-	74	53.2	-	-	64	63.4	-	-	0.117
Male	102	42.5	-	-	65	46.8	-	-	37	36.6	-	-	-
**Cadre**													
Physician	64	26.7	-	-	39	28.1	-	-	25	24.6	-	-	0.152
Nursing and midwifery	54	22.5	-	-	33	23.7	-	-	21	20.8	-	-	-
Laboratory health worker	46	19.2	-	-	31	22.3	-	-	15	14.9	-	-	-
Pharmaceutical personnel	29	12.1	-	-	12	8.6	-	-	17	16.8	-	-	-
Community support and public health worker	25	10.4	-	-	15	10.8	-	-	10	9.9	-	-	-
Other health workers (e.g. radiographers and anaesthetists)	22	9.2	-	-	9	6.5	-	-	13	12.9	-	-	-
**Healthcare facility level**													
Teaching hospital (level 1)	56	23.3	-	-	31	22.3	-	-	25	24.8	-	-	0.013
General hospital (level 2)	74	30.8	-	-	53	38.1	-	-	21	20.8	-	-	-
Community hospital (level 3)	110	45.8	-	-	55	39.6	-	-	55	54.5	-	-	-
**Confidence in COVID-19 vaccines**													
Disagree	42	17.5	-	-	18	13.0	-	-	24	23.8	-	-	< 0.001
Agree	154	64.2	-	-	105	75.5	-	-	49	48.5	-	-	-
Neutral	44	18.3	-	-	16	11.5	-	-	28	27.7	-	-	-
**COVID-19 vaccine recommendation**													
Not recommend	54	22.5	-	-	15	10.8	-	-	39	38.6	-	-	< 0.001
Recommend	186	77.5	-	-	124	89.2	-	-	62	61.4	-	-	-

Note: The characteristics of the participants are based on a survey conducted in ten Abidjan, Côte d’Ivoire, health facilities between May 2023 and June 2023. Statistically significant at *p* > 0.05.

IQR, interquartile range.

† , Individuals were classified as vaccinated if they had received a single dose of a one-dose vaccine, both doses of a two-dose vaccine or a booster dose.

Regarding the occupational distribution, 26.7% of participants were identified as physicians, 22.5% of participants were identified as nurses and midwives, 19.2% of participants were identified as pharmaceutical personnel, 10.4% of participants worked in community support and public health, and 9.2% of participants belonged to other health professions such as radiographers and anaesthetists. The participants were recruited from ten healthcare facilities distributed as follows: 23.3% from teaching hospitals, 30.8% from general hospitals and 45.8% from community hospitals (Table 1). These percentages corresponded to levels 1, 2 and 3 of the healthcare pyramid, that is, the primary care level, the secondary (regional facilities) care level and the tertiary (national referral) level.

### Participants’ willingness to get COVID-19 vaccine and vaccination status

Participants’ willingness to receive the COVID-19 vaccine was evaluated based on their age, gender, professional cadre and healthcare facility level. Age distribution showed significant differences (*p* < 0.001). Young individuals (< 35 years) were more represented among the unvaccinated (42.6%), while the age group (35–54 years) had 48.9% vaccinated HCWs. There was no significant association in vaccination regarding participants’ gender (*p* = 0.117) and professional cadre (*p* = 0.152). Regarding the relationship between health care facilities and vaccination status, a significant association was found (*p* = 0.013). However, there was a trend where unvaccinated individuals were more in higher-level healthcare facilities (Level 3) at 54.6% (Table 1).

Among all participants, more than half (53.3%, 95% CI: 51.4% – 64.2%) had completed full vaccination with either a single-dose or a two-dose COVID-19 vaccine. Approximately, 15.4% (95% CI: 11.1% – 20.6%) were partially vaccinated with one dose of a two-dose vaccine, and 4.6% (95% CI: 2.3% – 8.1%) had received a booster dose. Overall, 73.3% (95% CI: 67.3% – 78.8%) had received at least one dose of the COVID-19 vaccine. A small percentage (1.7%) did not disclose their vaccination status. A quarter of HCWs, 25% (95% CI: 19.7% – 31.0%), reported being unvaccinated. Overall, 42.1% of participants exhibited vaccine hesitancy.

**FIGURE 2:**
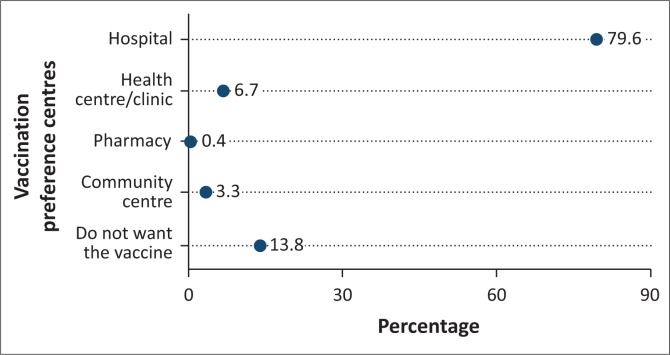
Vaccination centres preference of healthcare workers from ten health facilities in Abidjan, Côte d’Ivoire, between May 2023 and June 2023.

Figure 2 highlights the vaccination centres HCWs prefer for receiving the COVID-19 vaccine. Most HCWs (79.6%) expressed a preference for receiving the vaccine at a hospital, 6.7% at health centres or clinics and 3.3% at community centres. While 25% of respondents display reluctance towards vaccination, it is noteworthy that a considerable portion of respondents (13.8%) explicitly stated their unwillingness to receive the COVID-19 vaccine. Among vaccinated HCWs with at least one dose, 77.8% reported that it is easy to access vaccination services independently.

### Barriers and enhancers of COVID-19 uptake

Based on participants’ responses, various factors have been identified as potential barriers or enhancers of COVID-19 vaccine uptake. The confidence in the vaccine’s effectiveness against infectious diseases appears to be mixed among HCWs. Most participants somewhat agreed that vaccinating against COVID-19 and other infectious diseases, such as measles and tuberculosis, could reduce the risk of a person getting sick or dying. The agreement percentages were relatively modest at 33.8% and 47.5% for COVID-19 and infectious diseases, respectively.

**FIGURE 3:**
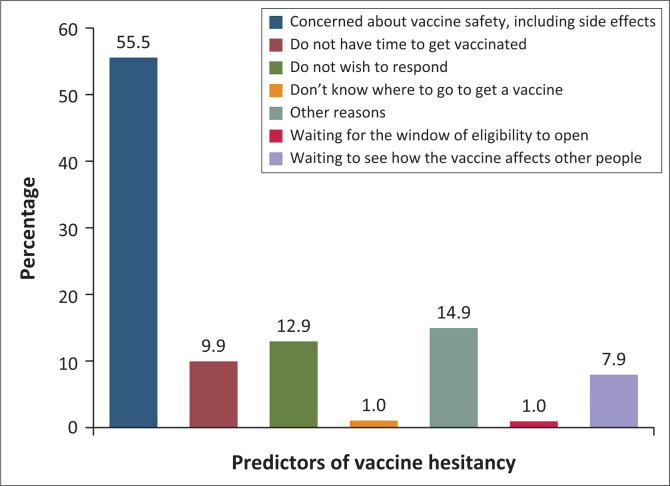
Reasons why healthcare workers were not yet fully vaccinated and/or boosted.

Figure 3 elucidates the factors contributing to why HCWs have not completed full vaccination and/or booster doses. The reasons identified in the figure shed light on the barriers or challenges this population faces in achieving complete vaccination coverage. The most common reason was their concern about the safety of the vaccines, including potential side effects (55.5%), followed by other reasons (14.9%), such as fear, pregnancy, having strong immunity or no interest.

**FIGURE 4:**
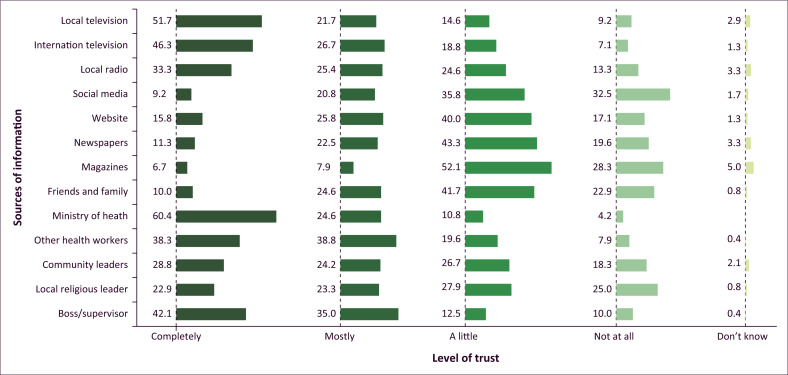
Trust in information sources about COVID-19 vaccination by healthcare workers from a cross-sectional survey carried out in ten healthcare facilities in Abidjan, Côte d’Ivoire, between May 2023 and June 2023.

Similar to what was mentioned above, unvaccinated respondents provided reasons for not wanting to receive an approved COVID-19 vaccine and/or booster. The primary concern cited was apprehension about serious side effects such as blood clots, neurological disorders or potential effects on motherhood (51.6%). This was followed by a lack of sufficient information about the vaccines before deciding to get vaccinated (15.6%) and a general mistrust of the currently approved vaccines (10.9%). Indeed, various sources of information were available to HCWs, highlighted in Figure 4, encompassing official government sources, media outlets, the internet and person-to-person communication. Notably, the sources considered the most trustworthy by HCWs were information from the Ministry of Health (MoH) at 60.4%, local television at 51.7% and international television at 46.3%. About 32.5% of respondents considered information on social media as not trustworthy.

Indeed, health workers’ lack of knowledge and confidence in licensed COVID-19 vaccines significantly affects their ability to address patients’ questions and provide advice on vaccination. Only 35.1% expressed confidence in their ability to answer patients’ questions about COVID-19 vaccine availability in Côte d’Ivoire. Nevertheless, more than half (55%) would still recommend COVID-19 vaccination to community members and patients prioritised to receive the vaccine.

Table 2 depicts the outcomes of the multivariable modified Poisson regression model, evaluating the association between demographic characteristics and the uptake of the COVID-19 vaccine with both crude and aPR, the 95% CIs and corresponding *p*-values. In the multivariable-adjusted model, age was a key determinant, with older individuals more likely to accept the COVID-19 vaccine, while pharmaceutical personnel showed a lower likelihood. Other factors, including gender, health facility level and vaccine confidence, did not have a statistically significant association. In detail, age showed a significant association with the vaccine uptake; HCWs aged 35–44 years were 1.60 times more likely to get the vaccine compared to those aged 22–34 years (aPR = 1.60, 95% CI: 1.16, 2.20). Healthcare workers aged 45–54 years had a higher likelihood of vaccination (aPR = 1.68, 95% CI: 1.20, 2.35) as those of 55–65 years (aPR = 1.78, 95% CI: 1.21, 2.62).

**TABLE 2:** Association between demographic characteristics and COVID-19 vaccine uptake among healthcare workers.

Characteristic	Crude PR	95% CI	Adjusted aPR	95% CI	Adjusted *p*-value*
**Age in years**					
22–34	ref	ref	ref	ref	-
35–44	1.66	1.20, 2.31	1.60	1.16, 2.20	**0.004**
45–54	1.64	1.14, 2.36	1.68	1.20, 1.35	**0.003**
55–65	2.25	1.57, 3.21	1.78	1.21, 2.62	**0.004**
**Gender**					
Female	ref	ref	ref	ref	-
Male	1.19	0.96, 1.47	1.18	0.95, 1.47	0.132
**Cadre**					
Physician	ref	ref	ref	ref	-
Nursing and midwifery	1.00	0.75, 1.34	0.99	0.75, 1.33	0.968
Pharmaceutical personnel	0.68	0.42, 1.09	0.7	0.47. 1.07	0.104
Laboratory health worker	1.11	0.83, 1.47	1.09	0.84, 1.40	0.518
Community support and public health worker	0.98	0.50, 0.74	1.14	0.82, 1.59	0.324
Other health workers	0.98	0.68, 1.43	0.76	0.44, 1.31	0.434
**Health facility level**					
Teaching hospital – level 1	ref	ref	ref	ref	-
General hospital – level 2	1.29	0.98, 1.71	1.38	1.07, 1.78	**0.012**
Community hospital – level 3	0.90	0.67, 1.22	1.15	0.86, 1.54	0.348
**Confidence in COVID-19 vaccines**					
Disagree	ref	ref	ref	ref	-
Agree	1.59	1.10, 2.29	1.05	0.71, 1.54	0.819
Neutral	0.85	0.50, 1.43	0.76	0.46, 1.25	0.279
**COVID-19 vaccine recommendation**					
Not recommend	ref	ref	ref	ref	-
Recommend	2.40	1.54, 3.74	2.11	1.30, 3.44	**0.003**

Note: The characteristics of the participants are based on a survey conducted in ten Abidjan, Côte d’Ivoire, health facilities between May 2023 and June 2023. Statistically significant at *p* > 0.05. Individuals were classified as vaccinated if they had received a single dose of a one-dose vaccine, both doses of a two-dose vaccine or a booster dose.

PR, prevalence ratio; aPR, adjusted prevalence ratio; CI, confidence interval; ref, reference category.

* , Significance of *p*-value set in bold.

Among professional categories, nursing and midwifery, and pharmaceutical personnel were less likely to receive the COVID-19 vaccine compared to physicians ([aPR = 0.99, 95% CI: 0.75, 1.33] and [aPR = 0.71, 95% CI: 0.47, 1.07], respectively). However, this association was not statistically significant.

Confidence in COVID-19 vaccines did not significantly impact COVID-19 vaccine uptake, whether individuals agree or remain neutral about their confidence. Finally, recommending COVID-19 vaccines was statistically significant (aPR = 2.11, 95% CI: 1.30, 3.44).

## Discussion

This investigation delved into the sentiments and concerns of Ivorian HCWs practising in Abidjan regarding authorised COVID-19 vaccines in the country across different demographics and professional cadres. The study’s findings revealed that there was a moderate uptake of COVID-19 vaccines among HCWs despite the availability of COVID-19 vaccines and the prioritisation of HCWs for vaccination in the country. More than half (53.3%) of HCWs completed full vaccination, suggesting reasonable success in the vaccination campaign among this category of the population, and the booster dose coverage (4.6%) indicated limited uptake follow-up protection. The COVID-19 vaccine uptake among HCWs in Côte d’Ivoire was low compared to global trends. This aligns with broader observations that vaccine uptake among HCWs in Africa is significantly lower than in other regions, with substantial variability influenced by country-specific factors and contexts.^12^

In comparison to other studies conducted in Africa, our findings on the COVID-19 vaccination uptake showed some similarities. A study examining COVID-19 vaccine hesitancy among Ethiopian HCWs found that 46.3% expressed hesitancy, primarily because of concerns about vaccine safety, efficacy and misinformation, which reflected our finding. Factors influencing hesitancy included younger age, lack of prior COVID-19 infection and limited trust in health authorities.^13^ In South Africa, where vaccine access and rollout infrastructure are relatively robust, a study reported vaccine hesitancy of 41% among HCWs in early 2022. Interstingly, factors like vaccine safety and fear of side effects, doubts about vaccine efficacy, low trust in health authorities, exposure to misinformation and younger age influenced vaccine refusal.^14^ This reflects similar challenges faced across Africa, such as concerns about side effects, limited trust in vaccines and accessibility issues.^15^ In this study, HCWs exhibited reluctance towards vaccination, primarily because of concerns about vaccine safety, potentially severe side effects, fear, pregnancy-related worries and perceived reliance on personal immunity. However, there was a conditional openness to receiving more information about vaccine safety before making a decision.

Our study found that 60% of unvaccinated individuals were female, which may reflect the higher proportion of women (57.5%) in the sample. In fact, the issue of vaccine hesitancy, particularly among women, has been explored in several studies.^12,16^ Research has shown that women, particularly in certain social and cultural contexts, are more likely to be hesitant about the COVID-19 vaccine compared to men. This trend was observed in different regions, including Africa, and may be linked to various factors such as perceived risks, social norms and misinformation.^12,16^ Gender differences in vaccine uptake are consistent with findings from studies in other regions, where women have expressed higher levels of reluctance or delayed vaccine adoption compared to men.^17^

In comparison to global studies, vaccine hesitancy among HCWs has been notably high among women.^18^ Research in countries such as Ethiopia and Canada has shown that female HCWs are more hesitant to take the COVID-19 vaccine compared to their male counterparts, although the reasons vary. These include concerns over safety and reproductive health.^19,20^

On a lighter note, most HCWs found it easy to access vaccines for themselves, and healthcare facilities were the preferred locations where they would prefer to get vaccinated. The finding aligned with the World Health Organization’s (WHO) recommendation to integrate COVID-19 vaccines into routine immunisation programmes, especially for high-risk groups such as HCWs, older adults and those with underlying conditions. This approach ensures sustained protection, particularly as COVID-19 transitions to an endemic phase.^21^

Misinformation and trust have been identified as factors for non-vaccine acceptance. In fact, the COVID-19 pandemic has attracted unprecedented media attention, and social networks have emerged as highly effective platforms for disseminating information about diagnostic and prevention measures.^8^ Additionally, they played a crucial role in shaping people’s behaviour in response to this public health threat, particularly regarding COVID-19 vaccine acceptance.^22,23^ In our findings, about 9.2% of healthcare professionals strongly believed in information from social media. Regrettably, the pandemic has seen social media as a conduit for spreading misinformation that has negatively shaped individuals’ behaviours regarding control measures.^8^ One should note that the emergence of severe acute respiratory syndrome coronavirus 2 (SARS-CoV-2) prompted pharmaceutical companies to invest in cutting-edge technologies for vaccine development. Because of the urgency presented by the virus, a number of these recently created vaccines did not undergo the entire validation process using state-of-the-art methods before being approved and authorised for use.^24^ This situation sparked concerns about the safety and efficacy of these vaccines, leading to a growing sense of mistrust in them.^25^

The issue of vaccine hesitancy is currently garnering unparalleled global attention, even among healthcare providers.^26^ This renders communities vulnerable to infectious diseases, leading to various outbreaks and ultimately depleting resources and claiming lives. The COVID-19 pandemic has raised significant concerns regarding the acceptance of vaccines among care providers. The global prevalence of COVID-19 vaccine hesitancy among healthcare providers was estimated to be 22.51%, with concerns about safety, efficacy and potential side effects cited as reasons for refusal.^27^ A systematic review undertaken in sub-Saharan Africa revealed that the vaccine hesitancy rate towards COVID-19 vaccines among healthcare providers in West Africa was estimated to be 52%, nearly two times higher than the refusal rate observed in this study. Factors such as negative attitudes towards vaccines, perceived low risk of COVID-19 infection and concerns about vaccine side effects emerged as predictors of vaccine hesitancy, aligning with our findings.^28^

Healthcare providers are crucial in managing and preventing vaccine-preventable diseases and the success of vaccination programmes. Not only are they at a higher risk as frontline workers, but their behaviours, attitudes, practices and beliefs regarding vaccines can also influence their attitude to recommend vaccines to the community they serve.^29^ Despite a COVID-19 vaccine hesitancy prevalence of 25% in our results, more than half of surveyed HCWs expressed their willingness to recommend the vaccine to their communities. This attitude underscores their recognition of vaccination as crucial in addressing the ongoing pandemic.

Our study has certain limitations that should be acknowledged. Firstly, the study was carried out in Abidjan, the capital city of Côte d’Ivoire, the epicentre of the pandemic in the country. Consequently, the vaccine hesitancy rate among HCWs may not represent the entire national scenario. Secondly, it is essential to notice that the study was conducted when the epidemic curve in the country was at its lowest, and the perceived threat of the disease may not have been as prominent in the collective consciousness at that time.

## Conclusion

The study revealed a significant rate of vaccine hesitancy, with 42.1% of participants remaining unvaccinated with women most likely to refuse to get vaccinated. Only 4.6% expressed interest in booster doses. The primary reasons for low vaccine uptake were concerns related to safety and potential side effects. Moreover, just over half of the surveyed HCWs were willing to recommend the vaccine to others in their community.

Efforts led by the MoH to educate HCWs on the safety of COVID-19 vaccines, coupled with local research into their effectiveness, can bolster vaccination rates and instil greater confidence among HCWs. These results underscore the critical importance of enhancing HCWs’ knowledge about vaccine safety. In addition, they highlight the necessity for local research initiatives to assess vaccine effectiveness. Emphasising the value of investing in pharmacovigilance programmes emerges as a crucial step in enhancing data on vaccine safety – a pivotal area requiring long-term investment for effective COVID-19 management.

## References

[1] Toor J, Echeverria-Londono S, Li X, et al. Lives saved with vaccination for 10 pathogens across 112 countries in a pre-COVID-19 world. Elife. 2021;10:e67635. 10.7554/elife.67635 34253291 PMC8277373

[2] John TJ, Plotkin SA, Orenstein WA. Building on the success of the expanded programme on immunization: Enhancing the focus on disease prevention and control. Vaccine. 2011;29(48):8835–8837. 10.1016/j.vaccine.2011.08.100 21971446

[3] Taylor MW. Introduction: A short history of virology. In: Viruses and man: A history of interactions. Cham: Springer; 2014. 10.1007/978-3-319-07758-1_1

[4] Cooper S, Betsch C, Sambala EZ, Mchiza N, Wiysonge CS. Vaccine hesitancy – A potential threat to the achievements of vaccination programmes in Africa. Hum Vaccin Immunother. 2018;14(10):2355–2357. 10.1080/21645515.2018.1460987 29617173 PMC6284499

[5] Yapi RB, Houngbedji CA, N’guessan DKG, et al. Knowledge, attitudes, and practices (KAP) regarding the COVID-19 outbreak in Côte d’Ivoire: Understanding the non-compliance of populations with non-pharmaceutical interventions. Int J Environ Res Public Health. 2021;18(9):4757. 10.3390/ijerph18094757 33946980 PMC8124153

[6] UNICEF. Côte d’Ivoire among the first countries to receive the first wave of COVAX vaccines [homepage on the Internet]. 2021 [cited 2024 Feb 19]. Available from: https://www.unicef.org/press-releases/cote-divoire-among-first-countries-receive-first-wave-covax-vaccines

[7] Storeng KT, De Bengy Puyvallée A, Stein F. COVAX and the rise of the ‘super public private partnership’ for global health. Glob Public Health. 2023;18(1):1987502. 10.1080/17441692.2021.1987502 34686103

[8] Calac AJ, Haupt MR, Li Z, Mackey T. Spread of COVID-19 vaccine misinformation in the ninth inning: Retrospective observational infodemic study. JMIR Infodemiology. 2022;2(1):e33587. 10.2196/33587 35320982 PMC8931848

[9] WHO. Statement on the fifteenth meeting of the IHR (2005) Emergency Committee on the COVID-19 pandemic [homepage on the Internet]. World Health Organization; 2023 [cited 2024 Dec 17]. Available from: https://www.who.int/news/item/05-05-2023-statement-on-the-fifteenth-meeting-of-the-international-health-regulations-(2005)-emergency-committee-regarding-the-coronavirus-disease-(covid-19)-pandemic

[10] Brewer NT, Chapman GB, Rothman AJ, Leask J, Kempe A. Increasing vaccination: Putting psychological science into action. Psychol Sci Public Interest. 2017;18(3):149–207. 10.1177/1529100618760521 29611455

[11] Greenwood M, Yule GU. An inquiry into the nature of frequency distributions representative of multiple happenings with particular reference to the occurrence of multiple attacks of disease or repeated accidents. J R Stat Soc. 1920;83(2):255–279. 10.2307/2341080

[12] Galanis P, Vraka I, Katsiroumpa A, et al. COVID-19 vaccine uptake among healthcare workers: A systematic review and meta-analysis. Vaccines. 2022;10(10):1637. 10.3390/vaccines10101637 36298502 PMC9610263

[13] Mohammed R, Nguse TM, Habte BM, Fentie AM, Gebretekle GB. COVID-19 vaccine hesitancy among Ethiopian healthcare workers. PLoS One. 2021;16(2):e0261125. 10.1371/journal.pone.0261125 34919597 PMC8682893

[14] Wiysonge CS, Alobwede SM, De Marie C, et al. COVID-19 vaccine acceptance and hesitancy among healthcare workers in South Africa. Expert Rev Vaccines. 2022;21(4):549–559. 10.1080/14760584.2022.2023355 34990311

[15] Mutombo PN, Fallah MP, Munodawafa D, et al. COVID-19 vaccine hesitancy in Africa: A call to action. Lancet Glob Health. 2022;10(3):e320–e321. 10.1016/s2214-109x(21)00563-5 34942117 PMC8687664

[16] Adeyanju GC, Engel E, Koch L, et al. Determinants of influenza vaccine hesitancy among pregnant women in Europe: A systematic review. Eur J Med Res. 2021;26(1):116. 10.1186/s40001-021-00584-w 34583779 PMC8477621

[17] Morales DX, Beltran TF, Morales SA. Gender, socioeconomic status, and COVID- vaccine hesitancy in the US: An intersectionality approach. Sociol Health Illn. 2022;44(6):953–971. 10.1111/1467-9566.13474 35500003 PMC9348198

[18] Desye B. Prevalence and determinants of COVID-19 vaccine acceptance among healthcare workers: A systematic review. Front Public Health. 2022;10:941206. 10.3389/fpubh.2022.941206 35968421 PMC9366855

[19] Dzieciolowska S, Hamel D, Gadio S, et al. Covid-19 vaccine acceptance, hesitancy, and refusal amomg Canadian healthcare workers: A multicenter survey. Am J Infect Control. 2021;49(9):1152–1157. 10.1016/j.ajic.2021.04.079 33930516 PMC8079260

[20] Yilma D, Mohammed R, Abdela SG, et al. COVID-19 vaccine acceptability among healthcare workers in Ethiopia: Do we practice what we preach? Trop Med Int Health. 2022;27(4):418–425. 10.1111/tmi.13742 35229414 PMC9115514

[21] WHO, UNICEF. Considerations for integrating COVID-19 vaccination into immunization programmes and primary health care for 2022 and beyond [homepage on the Internet]. World Health Organization; 2022 [cited 2024 Nov 28]. Available from: https://orbi.uliege.be/bitstream/2268/297838/1/Porignon_Integration%20of%20Covid19%20Vax_NIP_PHC_WHO%20UNICEF_2022.pdf

[22] Shaaban RS, Ghazy RM, Elsherif F, et al. COVID-19 vaccine acceptance among social media users: A content analysis, multi-continent study. Int J Environ Res Public Health. 2022;19(9):5737. 10.3390/ijerph19095737 35565132 PMC9101365

[23] Tsao SF, Chen H, Tisseverasinghe T, Yang Y, Li L, Butt ZA. What social media told us in the time of COVID-19: A scoping review. Lancet Digit Health. 2021;3(3):e175–e194. 10.1016/s2589-7500(20)30315-0 33518503 PMC7906737

[24] Defendi HGT, Da Silva Madeira L, Borschiver S. Analysis of the COVID-19 vaccine development process: An exploratory study of accelerating factors and innovative environments. J Pharm Innov. 2022;17(2):555–571. 10.1007/s12247-021-09535-8 33552310 PMC7851325

[25] Hasanzad M, Namazi H, Larijani B. COVID-19 anti-vaccine attitude and hesitancy. J Diabetes Metab Disord. 2022;22(1):1–4. 10.1007/s40200-022-01018-y 36373157 PMC9638374

[26] MacDonald NE, Dubé E. Unpacking vaccine hesitancy among healthcare providers. EBioMedicine. 2015;2(8):792–793. 10.1016/j.ebiom.2015.06.028 26425679 PMC4563149

[27] Biswas N, Mustapha T, Khubchandani J, Price JH. The nature and extent of COVID-19 vaccination hesitancy in healthcare workers. J Community Health. 2021;46(6):1244–1251. 10.1007/s10900-021-00984-3 33877534 PMC8056370

[28] Kigongo E, Kabunga A, Tumwesigye R, Musinguzi M, Izaruku R, Acup W. Prevalence and predictors of COVID-19 vaccination hesitancy among healthcare workers in sub-Saharan Africa: A systematic review and meta-analysis. PLoS One. 2023;18(7):e0289295. 10.1371/journal.pone.0289295 37506132 PMC10381063

[29] Schmid P, Rauber D, Betsch C, Lidolt G, Denker ML. Barriers of influenza vaccination intention and behavior – A systematic review of influenza vaccine hesitancy, 2005–2016. PLoS One. 2017;12(1):e0170550. 10.1371/journal.pone.0170550 28125629 PMC5268454

